# Correction to “Effect of spraying l‐menthol on peristalsis resumption during endoscopic submucosal dissection of gastric tumors”

**DOI:** 10.1002/jgh3.12908

**Published:** 2023-05-01

Akiyoshi Ishiyama, Ken Namikawa, Yoshitaka Tokai, Shoichi Yoshimizu, Yusuke Horiuchi, Toshiyuki Yoshio, Toshiaki Hirasawa, Tomohiro Tsuchida, Fumio Itoh, Junko Fujisaki. **Effect of spraying l‐menthol on peristalsis resumption during endoscopic submucosal dissection of gastric tumors.** *JGH Open*. 2021; **5**: 653–657.

The order of the authors' affiliations “*Department of Endoscopy, Cancer Institute Hospital Gastroenterology Center, Tokyo and ^†^Department of Gastroenterology, St. Marianna University School of Medicine, Kawasaki, Japan” was incorrect. The order of the authors' affiliations should have been: “*Department of Gastroenterology, St. Marianna University School of Medicine, Kawasaki, Japan and ^†^Department of Endoscopy, Cancer Institute Hospital Gastroenterology Center, Tokyo.”

The symbols representing the authors' affiliations were incorrect:

Akiyoshi Ishiyama*, Ken Namikawa*, Yoshitaka Tokai*, Shoichi Yoshimizu*, Yusuke Horiuchi*, Toshiyuki Yoshio*, Toshiaki Hirasawa*, Tomohiro Tsuchida*, Fumio Itoh^†^ and Junko Fujisaki*

The symbols should have appeared as follows:

Akiyoshi Ishiyama^*,†^, Ken Namikawa^†^, Yoshitaka Tokai^†^, Shoichi Yoshimizu^†^, Yusuke Horiuchi^†^, Toshiyuki Yoshio^†^, Toshiaki Hirasawa^†^, Tomohiro Tsuchida^†^, Fumio Itoh* and Junko Fujisaki^†^

In the ‘Methods’ section of the Abstract, the text, “We included 11**9** patients (127 lesions) in whom peristaltic movement resumed during ESD and l‐menthol was applied;….” was incorrect. This should have read: We included 11**6** patients (127 lesions) in whom peristaltic movement resumed during ESD and l‐menthol was applied;….” (bolded text indicates the error).

In the third column heading of Table 1, the text “Added l‐menthol 127 lesions, 11**9** cases” was incorrect. The text should have read: “Added l‐menthol 127 lesions, 11**6** cases” (bolded text indicates the error). The corrected Table 1 is reproduced below:

**Table 1:** Clinicopathological characteristics of the study population

		Added l‐menthol 127 lesions, 116 cases	Without l‐menthol 374 lesions, 366 cases	*P* value
M:F	344:138	84:32	260:106	0.775
Years		70.5 (43–84)	66.7 (36–89)	0.541
Lesion's size	15.8	14.4 (4–38)	16.6	0.792
Histology				
Intestinal	399	106	293	0.457
Diffuse	86	18	68	
Adenoma	16	3	13	
Depth				
M	440	119	321	0.019
SM	61	8	53	
Macroscopy				
0‐I	13	8	5	0.15
0‐IIa	118	35	83	
0‐IIc	273	68	205	
0‐IIa + IIc	42	6	36	
Others	55	10	45	
U:M:L	68:251:182	2 (0.8%):36 (28.8%):89 (71.2%)	66 (17.6%):215 (57.4%):93 (24.8%)	

M:F, male:female; M, mucosa; SM, submucosa; U:M:L, upper:middle:lower.

The authors apologize for these errors.

